# Effectiveness and Durability of Ustekinumab Therapy With or Without Immunomodulators for Ulcerative Colitis Patients in Japan

**DOI:** 10.1093/crocol/otac010

**Published:** 2022-03-21

**Authors:** Yasuhiro Aoki, Tomohisa Sujino, Takaaki Kawaguchi, Shinya Sugimoto, Fumie Shimada, Yusuke Yoshimatsu, Hiroki Kiyohara, Kosaku Nanki, Yohei Mikami, Kaoru Takabayashi, Naoki Hosoe, Haruhiko Ogata, Yasushi Iwao, Takanori Kanai

**Affiliations:** 1 Division of Gastroenterology and Hepatology, Department of Internal Medicine, Keio University School of Medicine, Tokyo, Japan; 2 Center for Diagnostic and Therapeutic Endoscopy, Keio University School of Medicine, Tokyo, Japan; 3 Center for Preventive Medicine, Keio University School of Medicine, Tokyo, Japan

**Keywords:** ustekinumab, immunomodulator, combination therapy, ulcerative colitis

## Abstract

**Background and Aims:**

The effectiveness and durability of ustekinumab therapy with or without thiopurine immunomodulators (IMs) for ulcerative colitis (UC) in real-world Asian, Japanese patients have not yet been elucidated.

**Methods:**

To evaluate the additive effects of IMs on ustekinumab, a retrospective cohort study of UC patients receiving ustekinumab with or without thiopurine IMs, azathioprine or 6-mercaptopurine, was conducted from March 2020 to August 2021. The primary endpoint was clinical remission or response rate at week 8. The secondary endpoints were clinical remission or response rates at weeks 24 and 52, the durability of each treatment, and adverse events.

**Results:**

Of the 50 patients with UC treated with ustekinumab, 42 were enrolled. Sixteen patients were treated with a combination of ustekinumab and an IM. The clinical response rates of all patients at weeks 8, 24, and 52 were 53.7%, 63.3%, and 42.9%, respectively. There was no significant difference in the clinical responses or remission rates between the combination therapy and monotherapy groups at weeks 8, 24, and 52. (50.0% vs. 56.0%, *P* = .757; 70.0% vs. 60.0%, *P* = .702; and 42.9% vs. 42.9%, *P* = 1.00, respectively). A Kaplan–Meier analysis showed no difference in IM use on the durability of ustekinumab treatment (log-rank test; *P* = .955).

**Conclusions:**

The response rate for Japanese UC patients is similar to previous reports based on American and European UC patients. There was no significant difference between the ustekinumab monotherapy group and the ustekinumab and IM combination group in the real world.

## Introduction

Ulcerative colitis (UC) is a chronic relapsing inflammatory bowel disease of unknown etiology. Although several new therapeutic agents have been developed in recent years, there remains no way to achieve a complete cure. Therefore, the ideal treatment for UC is the early induction and long-term maintenance of remission.

The efficacy of ustekinumab (UST) over placebo for UC has been demonstrated in the UNIFI trials. The clinical remission rates at 8 and 44 weeks after UST treatment were superior to those of the placebo group. The clinical remission rate at week 8 after UST induction treatment was 15% compared with 5% in the placebo group (*P* < .0001). Maintenance therapy clinical response rates at week 44 with subcutaneous (SC) injections of UST every 12 or 8 weeks were 38% and 44%, respectively (vs. placebo, *P* = .002 and *P* < .001, respectively).^1^ Besides the UNIFI clinical trial, the clinical remission rate in Japanese UC patients treated with UST in the real world is still unknown.

Thiopurine immunomodulators (IMs), including azathioprine and 6-mercaptopurine, are known not only as immunosuppressive agents for the treatment of UC but also to have an additional therapeutic effect on infliximab, a chimeric monoclonal antibody to tumor necrosis factor (TNF), used in the treatment of moderate-to-severe active UC.^2–4^ The benefit of IMs in combination with infliximab is derived from increasing the serum concentration of anti-TNF antibodies by inhibiting the production of anti-drug antibodies (ADAs).^5–8^ In contrast, IMs are known to increase the risk of severe infection and malignancy. Therefore, both the risks and benefits of the combination of IMs and biologics should be considered carefully.^6,9^

UST is a fully humanized monoclonal antibody against the p40 subunit of interleukin (IL)-12/23 that targets the Th1/Th17 inflammatory pathway. It has been proven that UST has long- and short-term efficacy in moderate-to-severe active UC^1,10^ and a low incidence of ADA production.^11^ In the UNIFI study, only 5.7% of ADA-positive patients received UST at one or more time points over a year. Moreover, the concomitant use of IMs with UST reduced the incidence of production of ADAs from 6.8% to 3.1%. On the other hand, it has been reported that the median serum concentration of UST was not influenced by the concomitant use of IM.^12^ Therefore, the benefit of the concomitant use of IMs and UST remains controversial. Phase III clinical trials of UST did not demonstrate a greater effectiveness of combination therapy with IMs than that of monotherapy.^1^

We aim to evaluate the real-world effectiveness of UST therapy in 8, 24, and 52 weeks as the treatment for Japanese UC. Moreover, we analyze the real-world effectiveness of UST therapy with or without IMs for UC patients in Japan.

## Materials and Methods

### Study Design and Patient Population

A single-center, retrospective cohort study was conducted at the (Keio: as for double blind review) University Hospital in Tokyo, Japan. The hospital database was searched for all patients with UC who had been treated with UST. Adult patients (≥16 years old) with active UC who received UST for induction therapy between March 2020 and August 2021 were included in this study. Patients with active UC were defined as having a partial Mayo score of 5–9 (maximum pMayo) or an endoscopic Mayo score of 2 or 3.

The observation period was from the beginning of treatment to the end of treatment until October 2021. Information about patient demographics, characteristics, medications, disease activity, and examination results was collected retrospectively by reviewing their medical records.

### Study Endpoints

The primary endpoint was the clinical response at week 8, which was evaluated using the partial Mayo score. Clinical remission was defined as a partial Mayo score of 0 or 1 and clinical response was defined as a decrease of at least 2 in the pMayo score from the baseline. The secondary endpoints were clinical remission or response at weeks 24 and 52, the durability of UST treatment, and adverse events.

### Statistical Analysis

To compare patient characteristics, 2 tests were used: the Fisher’s exact test for categorical data and the Mann–Whitney *U* test for continuous data. Summary statistical tables were prepared using frequencies and proportions for categorical data and means and standard deviations for continuous variables to evaluate patient background. The Kaplan–Meier method was used to estimate the efficacy of UST during the observation period and the differences between the curves were compared using the log-rank test. All *P* values were based on the 2-tailed hypothesis, and values less than .05 were considered statistically significant. All statistical analyses were performed using the JMP version 16.0.0 software (SAS Institute, Cary, NC, USA).

### Ethical Considerations

This study complied with the Declaration of Helsinki (revised in 2013) and the Guidelines for Medical Research Involving Human Subjects (Japanese Ministry of Health, Labor, and Welfare). The study protocol was reviewed and approved by the ethics committee of Keio (as for double blind review) University (no. 20150100). Data were collected anonymously.

## Results

### Patients

**Figure 1.**
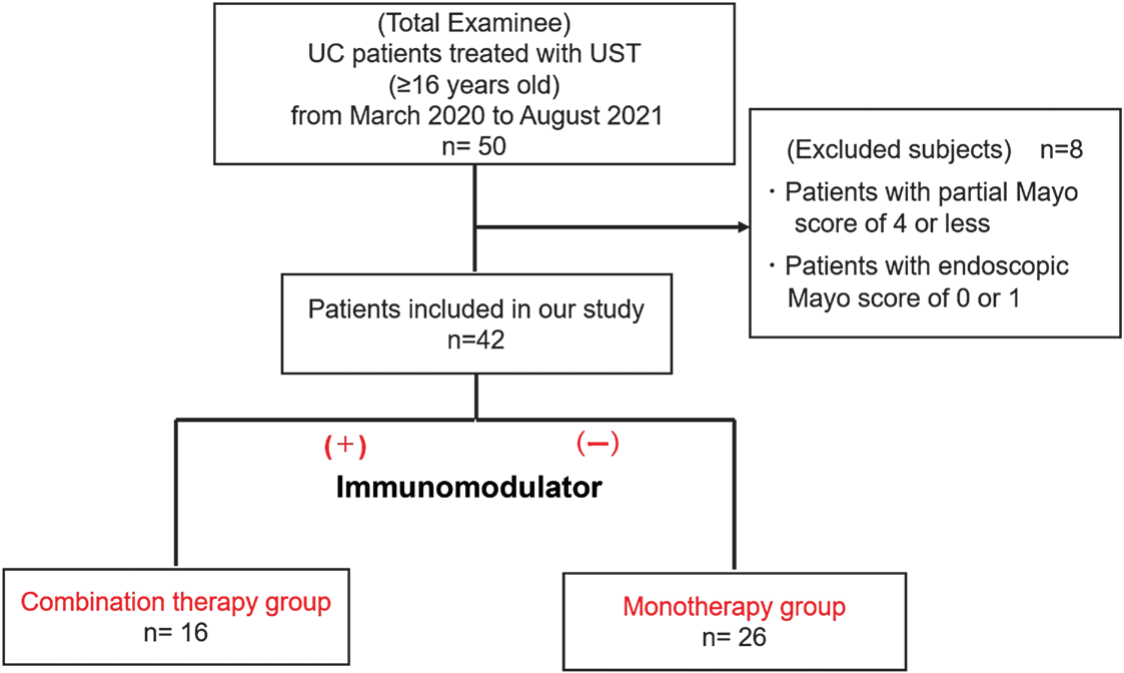
Study flow diagram. Fifty patients with ulcerative colitis treated with ustekinumab from March 2020 to August 2021 were enrolled. Among them, 42 patients were included in our study. These patients were divided into the combination therapy and monotherapy groups.

Fifty patients with UC who had been treated with UST were enrolled. Among these, 42 patients who met the inclusion criteria were included in the study (Figure 1). All the patients initially received UST by intravenous injection for the induction of remission, followed by a SC injection for the maintenance of remission. Forty-one patients were administered an SC dose of UST every 8 weeks and one patient was administered an SC dose of UST every 12 weeks. Of the 42 patients treated with UST, 16 received an IM concomitantly (9 patients received azathioprine and 7 patients received 6-mercaptopurine). Of these, 12 (75%) received a known Japanese therapeutic dose of an IM (azathioprine: more than 1.0 mg/kg/day or 6-mercaptopurine: more than 0.5 mg/kg/day); 87.5% (14/16) had used IM for more than 1 year and the other 2 patients had used IM for more  than 6 months before UST administration. Ten patients had used IM; however, they discontinued the use of IM owing to non-responsiveness or intolerance (5 cases of non-responsiveness, 3 of nausea, one of pancreatitis, and one of liver dysfunction). We included these 10 patients in the UST monotherapy group as they quit the use of IM at least 6 months before the initiation of UST. The remaining 16 patients had no experience with IM treatment.

### Baseline Characteristics

The baseline patient characteristics and medical histories are summarized in Table 1. When comparing the 16 patients treated with UST and IM combination therapy to the 26 patients who received UST monotherapy, there were no significant differences in patient background, past treatment history, or baseline disease activity.

**Table 1.** Baseline characteristics of patients initiating ustekinumab monotherapy or ustekinumab in combination with an immunomodulator.

	All, *n* = 42	IM (+), *n* = 16	IM (−), *n* = 26	*P* value
Age (years) (mean ± SD)	36.52 ± 14.32	41.56 ± 14.14	33.42 ± 13.80	.083^b^
Sex (male)	23 (54.8%)	8 (50.0%)	15 (57.7%)	.627^c^
Disease duration (years) (mean ± SD)	8.57 ± 7.64	11.25 ± 9.01	6.92 ± 6.30	.187^b^
History of bowel resection	1 (2.4%)	1 (6.3%)	0 (0%)	1.00^c^
Disease extension (E1/E2/E3^a^)	1/12/29 (2.4/28.6/69.0%)	1/6/9 (6.3/37.5/56.3%)	0/6/20 (0/23.1/76.9%)	.228^c^
Prior biologic exposures	34 (81.0%)	14 (87.5%)	20 (76.9%)	.688^c^
Partial Mayo score (mean ± SD)	4.71 ± 1.90	4.80 ± 2.04	4.67 ± 1.85	.883^b^
Endoscopic Mayo score 2/3	16/23 (38.1/54.8%)	7/8 (43.8/50.0%)	9/15 (34.6/57.7%)	.740^c^
Concomitant use of 5-ASA	37 (88.1%)	15 (93.8%)	22 (84.6%)	.633^c^
Concomitant use of PSL	8 (19.0%)	4 (25.0%)	4 (15.4%)	.454^c^
History of treatment				
5-ASA	41 (97.6%)	16 (100%)	25 (96.2%)	1.00^c^
Infliximab	21 (50.0%)	8 (50.0%)	13 (50.0%)	1.00^c^
Adalimumab	12 (28.6%)	6 (37.5%)	6 (23.1%)	.315^c^
Golimumab	4 (9.5%)	1 (6.3%)	3 (11.5%)	1.00^c^
Vedolizumab	26 (61.9%)	9 (56.3%)	17 (65.4%)	.745^c^
Prednisolone	38 (90.5%)	16 (100%)	22 (84.6%)	.280^c^
Tofacitinib	8 (19.0%)	2 (12.5%)	6 (23.1%)	.688^c^
Cyclosporine	3 (7.1%)	2 (12.5%)	1 (3.8%)	.547^c^
Tacrolimus	13 (31.0%)	6 (37.5%)	7 (26.9%)	.510^c^
Leukocytapheresis	9 (21.4%)	4 (25.0%)	5 (19.2%)	.711^c^

Abbreviations: 5-ASA, 5-aminosalicylic acid; IM, immunomodulator; PSL, prednisolone.

Montreal classification. E1: proctitis type, E2: left-sided colitis, E3: total colitis type.

Mann–Whitney *U* test.

Fisher’s exact test.

### Primary Analysis—8-Week Response

We analyzed 41 patients for 8-week response as the primary analysis due to 8-week clinical record. The clinical response or remission rate of all patients (*n* = 41) at week 8 was 53.7%.

**Figure 2.**
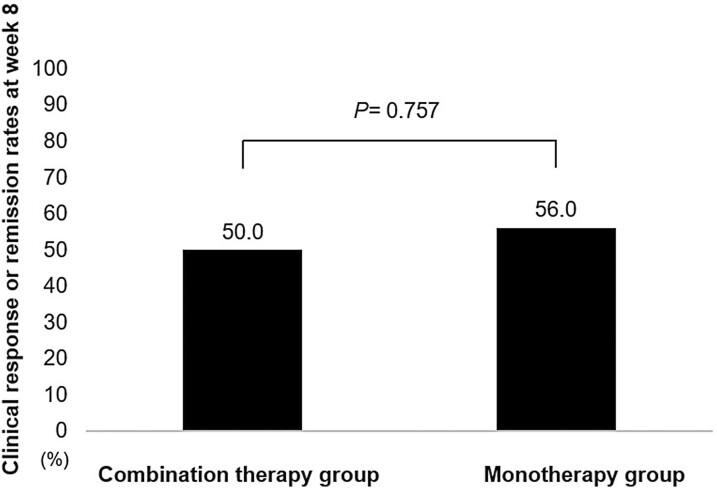
The primary endpoint for combination therapy compared with monotherapy with ustekinumab in patients with ulcerative colitis.

There was no significant difference in the clinical response or remission rate between the combination therapy group  (*n* = 16) and the UST monotherapy group (*n* = 25) at week 8 (50.0% vs. 56.0%; *P* = .757; Figure 2).

### Secondary Analysis

**Figure 3.**
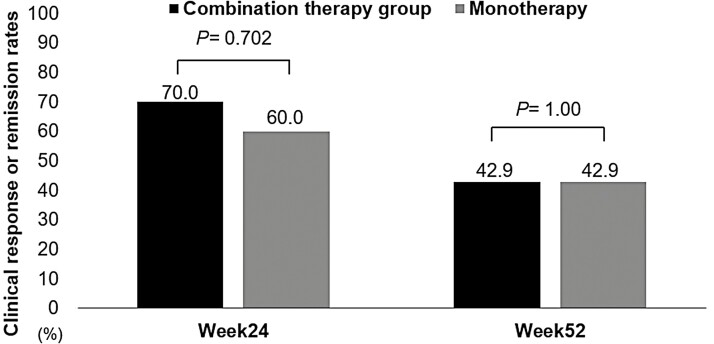
Clinical response or remission rate at weeks 24 and 52 for combination therapy compared with monotherapy with ustekinumab in patients with ulcerative colitis.

**Figure 4.**
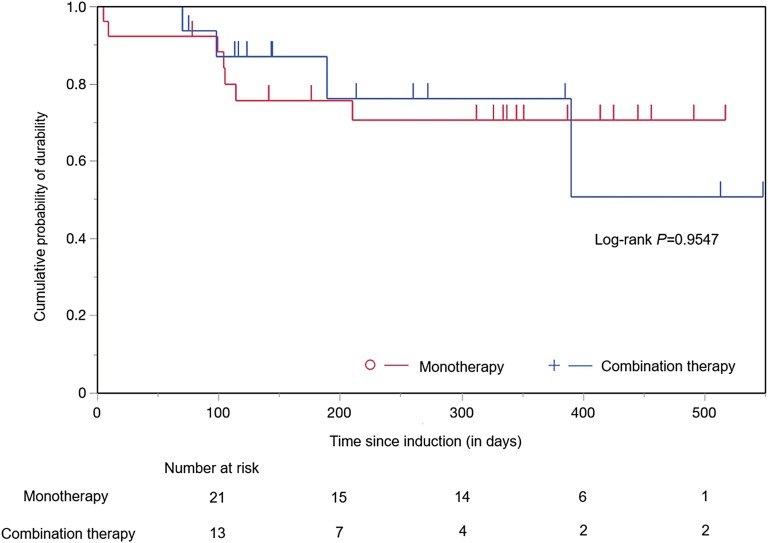
The Kaplan–Meier method was used to estimate the durability of ustekinumab monotherapy vs. combination therapy with a thiopurine immunomodulator.

The median observation periods for the combination and monotherapy groups were 211.5 (±SD, 157.1) days and 199.5 (±SD, 154.0) days, respectively. The clinical response or remission rates at weeks 24 (*n* = 30) and 52 (*n* = 21) were also comparable for the combination therapy and monotherapy groups (70.0% vs. 60.0%; *P* = .702 and 42.9% vs. 42.9%; *P* = 1.00, respectively; Figure 3; Table 2). The time to treatment discontinuation for UST combination therapy or monotherapy is shown in Figure 4. A Kaplan–Meier analysis showed that treatment with an IM had no impact on the durability of treatment with UST (log-rank test; *P* = .955).

**Table 2.** Comparison of clinical outcomes between ulcerative colitis (UC) patients with combination therapy and UC patients with monotherapy.

	Combination therapy % (*n*)	Monotherapy % (*n*)	*P* value
Clinical remission or response week 8	50.0% (8/16)	56.0% (14/25)	.757^a^
Clinical remission or response week 24	70.0% (7/10)	60.0% (12/20)	.702^a^
Clinical remission or response week 52	42.9% (3/7)	42.9% (6/14)	1.00^a^

Fisher’s exact test.

### Adverse Events

One patient in the UST monotherapy group discontinued treatment due to a skin rash, but there were no patients in the combination therapy group who discontinued UST due to adverse events. Another patient in the UST monotherapy group developed dermatitis of the palms, while one patient in the combination therapy group had a decreased white blood cell count, and his IM dosage was reduced.

## Discussion

We first report the effectiveness and durability of UST treatment using real-world data of Japanese UC patients. The effectiveness and safety of UST in UC patients were demonstrated in the UNIFI trial by comparison with placebo.^1^ Several reports have been published on the real-world efficacy of UST administration, especially in US and European patients. In the GETAID trial, Amiot et al^13^ conducted a retrospective efficacy and safety study of UST in patients with moderately to severely active UC. The clinical remission rate at 12–16 weeks was 40%, and steroid-free clinical remission rate was 35%; these are similar to our results.^13^ Hong et al^14^ in the United States reported that clinical remission rates at 3 and 12 months were 43% and 45%, respectively, in UC patients treated with UST. The steroid-free clinical remission rate was 35%, the endoscopic remission rate was 50%, and the mucosal and tissue endoscopic healing rate was 33% at 12 months. The median follow-up was 178 days (interquartile range: 57–483), which is comparable to our report.^14^ Chaparro et al^15^ reported 95 UC patients treated with UST during a median follow-up period of 31 weeks in the ENEIDA registry. The clinical remission rate at weeks 16, 24, and 52 were 53%, 39%, and 33% of patients, respectively.^15^ Compared with previous reports, our Japanese UC patient study shows the same efficiency as that observed in other countries. UST is administered at 8-week intervals for the treatment of UC in the United States and at 8- to 12-week intervals in Europe and Japan. In our study, almost all patients were given UST at 8-week intervals, which may explain why the results are similar to real-world data from the United States.

Combination therapy with biologics and an IM is widely used to increase and prolong the efficacy of biologic medicine by decreasing its immunogenicity.^8,16^ Recently, the “Treat to Target” strategy has become prevalent in the treatment of inflammatory bowel diseases^17^ but the loss of response to therapeutic agents remains a significant clinical concern.^18^ At the same time, patients who used combination therapy of biologics and an IM were concerned about cancer and infectious diseases, including COVID-19.

The role of IM combination therapy with anti-TNF therapy (infliximab and adalimumab) is well established.^7,8^ Combination therapy with azathioprine and infliximab increased the clinical and endoscopic remission rates when compared with monotherapy. Recently, a novel anti-α4β7 monoclonal antibody treatment using vedolizumab has been approved for both UC and Crohn’s disease (CD). The GEMINI 1 trial subgroup analysis showed no difference in patient outcomes between patients with CD who received vedolizumab monotherapy and those who received combination therapy.^19^ The IM-UNITI and CERTIFI studies did not show that the combination therapy of UST and an IM was superior to the outcomes of clinical responses of patients with CD.^20,21^

Hu et al^18^ reported a retrospective study of patients with CD or UC treated with combination therapy (UST + IM or vedolizumab + IM) or monotherapy with UST or vedolizumab alone. They enrolled 549 patients (263 patients with UC and 286 with CD) who were treated with maintenance therapy with vedolizumab and 363 patients (4 patients with UC and 359 with CD) who were treated with maintenance therapy with UST. Combination therapy with an IM and vedolizumab or UST did not show superiority to monotherapy in terms of clinical response and remission within 1 year. However, because they enrolled only 4 patients with UC who were treated with UST, the benefit of combination therapy for UST to treat UC patients remained unclear. This is the first real-world study to evaluate the efficacy of combination therapy of UST with an IM for induction therapy in Japanese patients with active UC. Our data suggest that the concomitant use of an IM with UST produced similar clinical remission rates to those observed for the use of UST monotherapy in Japanese patients with UC.

The benefits of an IM with biologics in combination therapy are believed to be due to several reasons. First, combination therapy with anti-TNF drugs reduces ADA formation, resulting in a higher concentration of biologics. One of the reasons for the lack of efficacy of UST combination therapy with an IM might be the fewer UST-induced ADAs. The UNITI trial and CERTIFI studies reported that 2.3% and 0.7%, respectively, of all patients expressed anti-UST antibodies.^20,21^ Azathioprine is a purine antagonist that is converted into 6-mercaptopurine in vivo,^22,23^ which inhibits leukocyte proliferation by interfering with nucleotide synthesis. It mainly inhibits T-cell proliferation but must be converted to an active metabolite for cells undergoing rapid cell division. UST is known to inhibit the generation of Th1 and Th17 cells by blocking the biological activity of IL12 and IL23 through their interaction with the cell surface receptor protein IL12Rβ1.^24^ Through this mechanism of action, UST can effectively neutralize IL12 (Th1) and IL23 (Th17)-mediated cellular responses.^25^ The additional effect of combination therapy may be limited because UST and an IM affect the same T cells. To support this idea, patients who respond well to UST express higher IL17 in the mucosa before treatment than those who do not.^26^

This study was conducted over a short period, and the effects of an IM may have been insufficient. Although there may not have been sufficient time for ADA formation, combination therapy may not be effective for short-term outcomes. Moreover, the total IM dose may have affected the outcome of this study, where 77.8% (7/9) of patients treated with azathioprine used >1 mg/kg/day and 71.4% (5/7) of patients treated with 6-mercaptopurine used more than 0.5 mg/kg/day. This was a lower IM dose than that generally used in Western countries. Genetic background, including NUDT15, might be involved in dose limitation in Asian patients with UC. Further studies are needed to assess the IM dose and its efficiency over a longer period.

However, there are advantages to this study conducted in a population of Japanese patients with active UC. Even though the duration of disease in the participants was longer in the combination therapy group than in the monotherapy group, this study provides proof that short-term monotherapy with UST was still effective. The effectiveness of UST with or without concomitant use of IM was shown in Japanese UC patients. In recent years, unnecessary treatments using IMs have been reduced because of increased risks of lymphoma, infection, and other side effects.^6,9^ In a study that combined all the clinical trials of UST for patients with IBD over 1 year, there was no difference in the incidence of serious adverse events, infections, major cardiovascular events, or malignancies (except for non-melanoma skin cancer) in 2574 patients who received UST and 1389 patients who received a placebo.^27^ These data suggest that UST-related adverse events are infrequent in the short term. In the present study, one patient was reported to have discontinued treatment while on monotherapy with UST due to a skin rash, which developed after the patient began UST treatment. The skin rash improved after UST was discontinued. However, none of the other patients discontinued therapy due to other adverse events.

There were several limitations to this study. First, this was a single-center retrospective study with a small sample size. Second, we did not observe the long-term efficacy of the combined use of UST and an IM. The clinical response rate was analyzed rather than the endoscopic remission. A multicenter, prospective, randomized controlled trial is needed to evaluate the benefits of combination therapy with a large number of samples over a long period. This study could not examine the patients when combination therapy was initiated. We observed that while all the patients in this study visited the hospital and received the medicine every 8–12 weeks, information regarding their adherence to the IM therapy was obscure. Furthermore, there was no observable additive effect of the clinical response to UST induction therapy in patients with UC.

## Conclusions

This is the first study to evaluate the effectiveness and durability of UST for UC patients in the real world in Japan. The effectiveness of UST in real-world Japanese UC patients was similar to the previous reports from other countries. Moreover, there was no significant difference in the effectiveness between the UST monotherapy group and UST plus IM combination therapy group.

## Data Availability

The data that support the findings of this study are available on request from the corresponding author.
